# Infection Induced Fetal Inflammatory Response Syndrome (FIRS): State-of- the-Art and Medico-Legal Implications—A Narrative Review

**DOI:** 10.3390/microorganisms11041010

**Published:** 2023-04-12

**Authors:** Elena Giovannini, Maria Paola Bonasoni, Jennifer Paola Pascali, Arianna Giorgetti, Guido Pelletti, Giancarlo Gargano, Susi Pelotti, Paolo Fais

**Affiliations:** 1 Unit of Legal Medicine, Department of Medical and Surgical Sciences, University of Bologna, Via Irnerio 49, 40126 Bologna, Italy; 2 Pathology Unit, Azienda USL-IRCCS di Reggio Emilia, Via Amendola 2, 42122 Reggio Emilia, Italy; 3 Department of Cardiac, Thoracic, Vascular Sciences and Public Health, University of Padova, Via Giustiniani 2, 35127 Padova, Italy; 4 Neonatal Intensive Care Unit, Azienda USL-IRCCS di Reggio Emilia, Via Amendola 2, 42122 Reggio Emilia, Italy

**Keywords:** fetal inflammatory response syndrome, placental pathology, causal link, medical liability, medico-legal compensation

## Abstract

Fetal inflammatory response syndrome (FIRS) represents the fetal inflammatory reaction to intrauterine infection or injury, potentially leading to multiorgan impairment, neonatal mortality, and morbidity. Infections induce FIRS after chorioamnionitis (CA), defined as acute maternal inflammatory response to amniotic fluid infection, acute funisitis and chorionic vasculitis. FIRS involves many molecules, i.e., cytokines and/or chemokines, able to directly or indirectly damage fetal organs. Therefore, due to FIRS being a condition with a complex etiopathogenesis and multiple organ dysfunction, especially brain injury, medical liability is frequently claimed. In medical malpractice, reconstruction of the pathological pathways is paramount. However, in cases of FIRS, ideal medical conduct is hard to delineate, due to uncertainty in diagnosis, treatment, and prognosis of this highly complex condition. This narrative review revises the current knowledge of FIRS caused by infections, maternal and neonatal diagnosis and treatments, the main consequences of the disease and their prognoses, and discusses the medico-legal implications.

## 1. Introduction

Fetal inflammatory response syndrome (FIRS) is defined as a pathologic condition of systemic fetal inflammation. It may occur after direct fetal exposition to inflammation in the amniotic fluid or through placental-fetal blood circulation, resulting in chorioamnionitis (CA), the inflammation of amnion and chorion. FIRS may also be induced by non-infectious processes with increased levels of fetal plasma cytokines [1]. The latter causes will not be analyzed in depth in this current review, as our focus will be on FIRS determined by infections. The reader should then refer to the specific diseases subsequently mentioned.

Interest in FIRS has been progressively growing in the last few years. Indeed, conclusive evidence regarding early diagnosis and treatment is lacking, despite its high morbidity and mortality rate. Therefore, it may frequently result in litigation and claims for compensation.

The aim of this study is to provide a narrative review of the literature, focusing on infection-induced FIRS, the state-of-the-art research on etiopathogenesis, prenatal and postnatal diagnosis and treatment, and potential newborn consequences, also taking into consideration the medico-legal implications.

## 2. Materials and Methods

A literature search in the electronic databases PubMed, Scopus and Web of Science was conducted using a combination of free text protocols as follows: (fetal inflammatory response syndrome OR FIRS OR chorioamnionitis OR funisitis) AND (etiopathogenesis OR diagnosis OR treatment OR consequences OR medico-legal OR post-mortem examination OR autopsy). The research included articles published between January 2000 and December 2022.

Articles related to FIRS etiopathogenesis, diagnosis, treatment, postnatal consequences, and medico-legal implications (autopsy and/or histological findings, fetal outcome at birth, ethical or compensation issues) were included in the review. Reports written in English, French, German, Spanish, and Italian were considered.

## 3. Results

**Figure 1:**
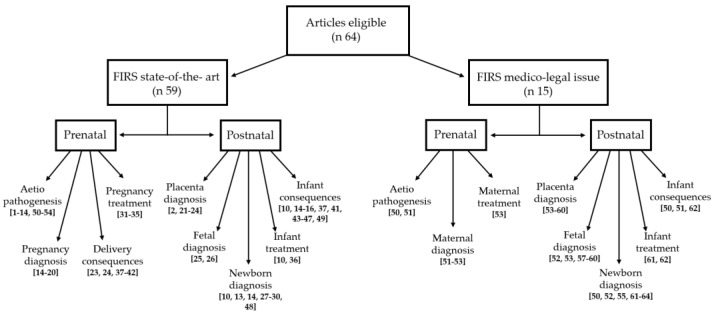
Flow chart of eligible articles found for narrative review.

A total of 870 references were identified. Sixty-four (64) articles were eligible according to our inclusion criteria [1,2,3,4,5,6,7,8,9,10,11,12,13,14,15,16,17,18,19,20,21,22,23,24,25,26,27,28,29,30,31,32,33,34,35,36,37,38,39,40,41,42,43,44,45,46,47,48,49,50,51,52,53,54,55,56,57,58,59,60,61,62,63,64]. Forty-nine concern prenatal/postnatal related themes [1,2,3,4,5,6,7,8,9,10,11,12,13,14,15,16,17,18,19,20,21,22,23,24,25,26,27,28,29,30,31,32,33,34,35,36,37,38,39,40,41,42,43,44,45,46,47,48,49] and 15 concern medico-legal issues [50,51,52,53,54,55,56,57,58,59,60,61,62,63,64] (Figure 1).

## 4. Discussion

### 4.1. Definition of FIRS

FIRS represents the fetal immune response to infection or injury mediated by the release of cytokines and chemokines, leading to multiorgan impairment, neonatal mortality and morbidity [1].

FIRS is often caused by an infectious process, such as microbial invasion of the amniotic cavity, placental membranes or parenchyma, resulting in chorioamnionitis (CA) and progressive fetal inflammation [2,10].

Non-infectious processes can also increase cytokine release favouring FIRS, i.e., preterm delivery, meconium peritonitis, and immunoreactive reactions [64,65,66,67,68,69].

### 4.2. Aetiopathogenesis of FIRS

FIRS is a complex pathophysiologic condition characterized by systemic or local inflammation in different organs due to elevation of fetal plasma cytokines, in particular the pro-inflammatory types (TNF-alpha, IL-1, and IL-6). The latter (IL6) were elevated in postmortem premature brains with periventricular leukomalacia (PVL) that had been exposed to infections, compared to PVL without infection exposure [8,9,10].

Cytokines are peptides or glycoproteins designated to control the innate and adaptive immune responses through intracellular communication. IL-6 is the major mediator of the acute phase response to tissue injury and can be measured in peripheral circulation [1]. Increased pro-inflammatory cytokines induce the activation of local inflammation in different fetal organs, including the brain, heart, lungs, skin, hematopoietic system, kidneys, adrenal glands and thymus, leading to further tissue damage [10]. Pro-inflammatory cytokines are particularly harmful for oligodendrocytes and neurons. Furthermore, pro-inflammatory cytokines alter brain development via microglial activation, which plays a key role in newborn brain injuries. The integrity of the blood-brain barrier is compromised in the setting of inflammation, resulting in increased permeability to peripherally generated inflammatory cells and other cytotoxic proteins [11,12,13].

Most often, FIRS is caused by a microbial infective process that may follow ascending, hematogenous, or transabdominal pathways. The most frequent ascending route is from the perineum, cervix, and vagina. Escherichia coli is the most common pathogen. However, other agents can be involved, such as Candida, Actinomyces, Prevotella bivia, Corynebacterium sp., Peptostreptococcus magnus, multiple Streptococcus species, Mycoplasma sp., Ureaplasma urealyticum, and Treponema Pallidum [2,4]. Hematogenous spreading may occur during maternal sepsis or temporary bacteremia, which also occurs in dental infections. The last condition may explain the role of Fusobacterium in inducing CA and premature ruptures of membranes [4,5,6]. Listeria monocytogenes typically infects the intervillous space through the bloodstream [5]. Contiguous contamination may spread from adjacent infected sites, for instance, the fallopian tubes, peritoneum, bladder, appendix, and intestine. Amniocentesis, being an invasive procedure, may determine direct amniotic fluid adulteration [4]. The duration of labour and presence of membrane ruptures may also represent significant risks of infection. Other obstetric factors include multiple intrapartum digital vaginal examinations, cervical insufficiency, an intracervical balloon catheter, and the presence of genital tract pathogens (e.g., sexually transmitted infections, group B Streptococcus, bacterial vaginosis) [5,6].

Non-infectious processes can also increase cytokine release, especially IL-6, resulting in FIRS [64,65,66,67,68,69].

This cytokine has been reported as being elevated in umbilical cord blood in newborns with meconium-stained amniotic fluid, as well as in plasma of anemic fetuses with Rhesus alloimmunization [64,65,66,67,68]. 

Moreover, preterm labour was found to be associated with elevated placental cytokine release, confirmed by IL-6 expression in newborn serum. In particular, placental cells from uninfected women delivering preterm produced significantly larger amounts of cytokines than cells from nonlaboring women at term [69].

### 4.3. Diagnosis of FIRS

#### 4.3.1. Prenatal Diagnosis (Diagnosis in Pregnancy)

During pregnancy, diagnosis of FIRS is primarily based on clinical signs and symptoms. Clinical signs suggestive of inflammation in the mother include fever, malodorous and purulent-appearing amniotic fluid, uterine tenderness and/or tachycardia [14,15]. With regard to laboratory tests, there is evidence of elevated maternal white blood cell count, increased C-reactive protein (CRP) and, in severe cases, bacteremia [14,16]. The gold standard for maternal diagnosis is represented by invasive procedures. Amniocentesis can be performed to assess the presence of leukocytes in the amniotic fluid; or IL-6 levels in cervicovaginal secretions [17].

The main sign of fetal distress is tachycardia, detectable via ultrasound [14,15]. Moreover, ultrasound measurement of thymus dimensions might constitute a reliable marker of FIRS [26].

A presumptive diagnosis can be made in pregnant women with fever associated with one or more of additional observations, such as fetal tachycardia, maternal leukocytosis and cervical purulent-appearing fluid. A confirmed diagnosis of intraamniotic infection can be made with all the above criteria for suspected intraamniotic infection associated with one or more findings of confirmed infection (e.g., positive amniotic fluid culture, low glucose level in amniotic fluid, a high count of leukocytes in amniotic fluid, histopathologic evidence of infection or inflammation) [18,19].

As previously mentioned, “*Triple I*” is diagnosed when maternal fever is present associated with one or more of the following signs: fetal tachycardia, maternal leukocytosis, purulent discharge from the cervical os and biochemical or microbiologic evidence of amniotic fluid infection [20].

#### 4.3.2. Postnatal Diagnosis

##### Placental Diagnosis

Following delivery, macroscopic and microscopic analyses of the placenta can confirm the prenatal diagnosis of FIRS, providing additional information on its extension and severity. Acute funisitis and chorionic vasculitis represent the histological equivalent of FIRS, also defined as fetal inflammatory response (FIR). The first is due to fetal neutrophils within the umbilical vessels with or without extension to the Wharton’s jelly. The second is acute inflammation within the wall of chorionic vessels. Fetal neutrophils migrate from the circulation through the endothelium and towards the amniotic fluid cavity, chemotactically attracted by the ongoing infection [2,21,22]. Histological FIR is highly associated with a fetal plasma concentration of IL-6 > 11 pg/mL, progressively increasing according to its severity [23].

Staging and grading have been defined by Redline et al. [24]. Staging defines the neutrophilic extension into the umbilical cord or chorionic plate, and grading refers to its severity.

Stage 1 corresponds to intramural umbilical phlebitis or chorionic vasculitis and is a mild-moderate neutrophilic infiltration.

Stage 2 involves one or both the umbilical arteries ± extension to the Wharton’s jelly; or phlebitis with neutrophil presence within the jelly; or intramural inflammation of all the vessels with or without the jelly’s involvement. Stage 2 defines neutrophilic aggregation within chorionic and/or umbilical vessels ± fading or degeneration of the vascular smooth muscle cells [24].

Stage 3 represents necrotizing funisitis or concentric umbilical perivasculitis.

##### Fetal Diagnosis

In cases of fetal death, autopsies and histological examinations are the gold standards to detect FIRS. In fetuses, when CA is the recognized cause of death, it is common to find aspirated polymorphonuclear leukocytes in the lungs, stomach, intestines, or internal ears. During the mid-trimester of gestation, extramedullar hematopoietic activity is predominantly located in the hepatic tissue; CA induces significant hematopoiesis extension into the lungs, pancreas, and adrenal glands. Neonatal sepsis and CA lead to morphologic modifications and shrinkage of the thymus and spleen [25]. Lastly, in the context of histological CA, fetal dermatitis can be a clinical sign, characterized by infiltration of neutrophils, lymphocytes, and histiocytes [26].

##### Newborn Diagnosis

In newborns with suspected FIRS, signs of cerebral hypoxia are investigated first.

In the newborn, evidence of infection can be detected radiologically in the brain at the level of white matter, thalamus, basal ganglia, cortex, brain stem, and cerebellum [27,28]. Cerebral ultrasounds rapidly identify focal periventricular leukomalacia, as macroscopic cystic changes due to necrosis of the deep cerebral white matter. Magnetic Resonance Imaging (MRI) is the gold standard for diffusing periventricular leukomalacia and other FIRS related lesions, such as isolated deep grey or white matter injuries, and intraventricular hemorrhages [10,13,28,29,30].

Brain injuries progressively evolve into cerebral atrophy, which is easily identified by MRI and characterized by reduced white matter volume and ventricular enlargement [13,29].

The neuroimaging findings of brain injuries caused by FIRS are not specific and overlap with those of hypoxic-ischemic injury [14,28].

### 4.4. Treatment of FIRS

#### 4.4.1. Prenatal Treatment (Pregnancy Treatment)

FIRS is one of many possible complications of CA during pregnancy, and therefore it is crucial to prevent infection of the membranes by identifying and treating associated risk factors. One of the main risk factors for CA is premature ruptures of membranes (PROM) [31]. Researchers continue to debate whether or not antibiotic treatment of patients with PROM will subsequently prevent CA and thus prevent its adverse neonatal outcomes. In cases of PROM, group B streptococcal prophylaxis should be given based on prior culture results or, if not performed, intrapartum risk factors only. In cases where GBS group B streptococcal prophylaxis is not required, there is insufficient evidence to justify the routine use of prophylactic antibiotics with premature ruptures of membranes at term [32]. A meta-analysis found that inducing labour reduced the time between the rupture of membranes and birth and consequently the rates of CA, endometritis, or both, as well as admission to a neonatal intensive care unit (NICU) [33].

Non-reassuring fetal status, clinical CA, and significant abruptio placentae are clear indications for delivery in patients at 34 weeks of gestation or greater [32].

Management of preterm premature rupture of membranes (PPROM) generally consists of hospital admission with periodic assessments for infection, abruptio placentae, umbilical cord compression, fetal well-being, and labour. A rising temperature may indicate intrauterine infection. Prompt diagnosis of CA in preterm pregnancy requires a high index of suspicion because early signs and symptoms may be subtle. Serial monitoring of white blood cells and other markers of inflammation have not been proven to be useful and are nonspecific when there is no clinical evidence of infection, especially if antenatal corticosteroids have been given. Treatment of acute CA includes antimicrobial agents, antipyretics, acceleration of delivery and management of additional symptoms [32,33,34].

Despite the fact that CA is common, there is limited evidence to support one specific antibiotic regimen over another. The most commonly employed antibiotics are ampicillin for coverage of Gram-positive organisms, gentamicin for coverage of Gram-negative bacteria, and clindamycin for additional coverage of anaerobes in the event of a caesarean section. A lower risk of postpartum endometritis has been observed in women who had a vaginal delivery and were previously treated with clindamycin in addition to ampicillin and gentamicin; there were no differences in women delivering by caesarean section. In case of caesarean section delivery and CA, the recommendation was to add anaerobic coverage. Despite the paucity of evidence, the current standard of practice is to initiate antibiotic treatment promptly once the diagnosis of CA has been made. Although delivery should be expedited, caesarean section remains reserved for the usual obstetric indications [32,34].

According to the selected literature, antibiotic or antiviral treatments have no benefit in regard to avoiding or limiting brain injuries [10].

#### 4.4.2. Postnatal Treatment (Infant Treatment)

Immediately after delivery, postnatal therapy aims to prevent and treat the effects of FIRS, especially on the brain. The first therapeutic strategy for FIRS is hypothermia, exerting neuroprotection through decreasing metabolism, reducing free radical production, inhibiting apoptosis, and slowing down activation of the immune response. The use of therapeutic hypothermia has demonstrated a significant decline in the rate of death or severe disability at 18 months, with improved neurocognitive outcomes at 6–7 years of age [10,36,62].

Experimental studies on the use of IL receptor antagonists (IL-1RA) and glucagon-like peptide-1 receptor agonists (GLP1R) demonstrated their key role in regulating the immune response, lowering systemic cytokine concentration. Finally, the administration of magnesium sulphate may have anti-inflammatory properties during pregnancy [10,36].

### 4.5. Consequences of FIRS

#### 4.5.1. Prenatal Consequences

##### Preterm Delivery

Preterm labour and delivery are the primary adverse outcomes of FIRS, especially in cases of necrotizing funisitis [24,37,38,39,57]. Preterm labour in the setting of infection results from the action of maternal and/or fetal pro-inflammatory cytokines in response to the intra-amniotic infection [23]. Due to baby hypotonia, FIRS can lead to traumatic complications during labour, such as brachial plexus palsy, without undue force [40,61]. Almost 50% of pregnancies associated with clinical or histologic CA terminate at preterm gestation with an increased rate of neonatal morbidity, especially neurodevelopmental impairment [12,41,42]. Multivariate analyses showed that FIRS was an independent predictor of severe neonatal morbidity, after adjustment for gestational age, the obstetrical cause of preterm delivery (preterm labour or preterm premature ruptures of membranes), clinical CA, presence of microorganisms in the amniotic cavity, and levels of IL-6 in the amniotic fluid [23].

##### Fetal Death

Fetal death is due to activation of inflammation and dysregulation of immunity as a result of diffuse placental infection. This condition can result in severe damage to all fetal organs, particularly the brain [8,9,11,12,13]. Furthermore, FIRS can induce a decrease in oxygenation and/or blood flow, leading to hypoxic-ischemic encephalopathy. This damage represents the most severe sequelae of FIRS, possibly followed by intrauterine fetal death. [14].

#### 4.5.2. Postnatal Consequences

##### Infant Neurological Damage

FIRS increases the risk of neonatal encephalopathy (NE) in term infants by a factor of twelve [10,43].

The fetal and neonatal brain is highly susceptible to inflammation and oxidative stress as it continues developing during the third trimester, early postnatal period, and the first few years of life [10]. The severity of inflammation highly correlates with cerebral damage, and the most severe cases are related to necrotizing funisitis and severe chorionic vasculopathy [43,44,45,46].

NE is a clinical syndrome that occurs in the first days of life, characterized by low consciousness or convulsions, breathing difficulties, such as difficulty starting or maintaining the respiration, hypotonic muscles, and abnormalities in the nerve reflexes [14]. NE can be the result of various disorders: coagulation anomalies, infections, autoimmune conditions, metabolic, and genetic diseases. On the whole, the clinical signs of NE are not etiologically specific [15,36,61,62]. NE is associated with high morbidity and mortality rates, as well as long-term disabilities that include cerebral palsy, cognitive impairment, epilepsy, blindness, deafness, and speech/language disorders [44,57]. NE is a predictor of possible severe neurological disabilities and up to 20% of affected infants die in the neonatal period. Infants with moderate to severe NE develop cerebral palsy in one third of cases [57].

Cerebral palsy is a syndrome that combines permanent motor and postural disorders, leading to motor disabilities of spastic, dyskinetic or ataxic type(s), and is often associated with sensory and cognitive impairments [16,52]. Cerebral palsy is also associated with decreased life expectancy, especially in patients with significant intellectual disabilities [41]. Among the non-hypoxic causes of cerebral palsy, the role of preterm intrauterine infection remains controversial. However, prevention of perinatal infection, a recognized risk factor for cerebral damage, represents a useful tool in hampering cerebral palsy development [14,63].

Finally, cerebral inflammation increases the risk of neurodevelopmental impairment during childhood and adulthood [43,44,45,46].

##### Other Infant Organs Damage

Although neurological disorders are the most significant complications of FIRS, this condition can damage other organs and systems. FIRS can cause respiratory distress syndrome due to increased secretion of cortisol, accelerating fetal lung maturation and surfactant production. Furthermore, FIRS has been associated with more severe and persistent pulmonary hypertension and later bronchopulmonary dysplasia. Cardiac complications include fetal heart rate disturbances, changes in diastolic ventricular function, reduction in cardiomyocyte numbers, and persistent patent ductus arteriosus [10,47,48].

Spontaneous intestinal perforation and necrotizing enterocolitis are more frequent in newborns with placental CA. Abnormal cortisol-to-dehydroepiandrosterone ratios and hematological alterations (neutrophilia, neutropenia, elevated nucleated red blood cell counts, and thrombocytopenia) have been reported in infants with FIRS [1,10,26].

A meta-analysis of observational studies has shown that FIRS was associated with a higher frequency of adverse outcomes when compared to neonates without FIRS [49].

More specifically, early-onset sepsis, respiratory, neurologic, and cardiac disorders were described. Moreover, in preterm neonates, FIRS was significantly and independently associated with an increased risk of retinopathy of prematurity and neonatal systemic inflammatory response, presenting as clinically suspected neonatal sepsis with negative blood and cerebrospinal fluid cultures [37,38,47].

### 4.6. Medico-Legal Implications of FIRS

FIRS is a condition with an extremely complex etiopathogenesis and a high morbidity and mortality rate. Therefore, it can frequently give rise to litigation and requests for compensation [1,50]. Indeed, families desperately seek the reason for their baby’s illness or death and if any medical procedure had been correctly applied [50].

As previously discussed, the pathophysiology of FIRS is currently still being studied and understanding the series of events that resulted in fetal injury remains extremely complicated [8]. Considering these issues, the forensic pathologist might adopt a very prudent approach for the identification of the aetiopathogenesis of FIRS, bearing in mind that this is often a late diagnosis and that defining a precise timing of infection is usually cumbersome. Indeed, timing of the injury is essential, especially the onset, in order to ascertain whether or not a specific condition could have been clinically identified and was missed [8].

During pregnancy, identifying the timing of FIRS onset is challenging. Maternal symptoms are nonspecific (e.g., maternal fever, tachycardia or leukocytosis, or fetal tachycardia) and may just lead to a presumptive diagnosis. On the other hand, diagnosis confirmation may require invasive investigations associated with complications (e.g., amniocentesis) [19].

Of note, in almost 75% of cases, chorioamnionitis is histologically present with no accompanying maternal clinical signs and/or symptoms of infection [2,14,58]. Especially in this latter condition, the diagnosis of FIRS may be performed belatedly when the newborn is already compromised or even after death.

The authors highlight that the ex-ante reconstruction of the ideal conduct, in each specific case, according to clinical setting and time condition, remains difficult due to uncertainty about FIRS diagnosis. Thus, FIRS is highly likely identified through histological placental examination, or in case of stillbirth, at fetal autopsy.

During legal proceedings, the forensic pathologist may be asked to specify the timeframe of placental or fetal lesions due to CA and FIR. Precise timeframes are usually impossible to define on the basis of placental changes. Additionally, the pathologist may be questioned as to whether or not a specific placental lesion directly caused the adverse clinical outcome. Caution is required in this evaluation, as a direct cause-and-effect relationship is barely supported by current scientific basis [57].

A thorough review of the medical records available is mandatory to reconstruct the relation between FIRS and clinical manifestations to correctly investigate healthcare flaws [50]. All the available maternal and neonatal information should be obtained in order to find unacknowledged clinical, laboratory or radiological findings, which must be interpreted by integrating placental histological features [50,57,60]. Evaluation of the medical records must include the identification of risk factors that increase the probability of infection and therefore FIRS, especially PROM, but also multiple intrapartum digital vaginal examinations, cervical insufficiency, intracervical balloon catheters, and the presence of genital tract pathogens [5,6,54,55].

The ex-ante reconstruction of the ideal medical conduct also remains problematic considering the uncertainty surrounding therapy and prognosis of FIRS. As previously discussed, there are no proven methods to prevent or treat FIRS during pregnancy and it is still unclear if antibiotic or antiviral administration are beneficial in preventing adverse neonatal outcomes and brain damage [19,32,35,36,64]. In FIRS, it is not possible to affirm with certainty that antibiotic therapy, even when indicated, could always have avoided infection and its consequences with high probability, because on this issue, the data from the literature is still insufficient [50]. Moreover, similarly to prenatal treatment during pregnancy, postnatal therapeutic interventions currently have limited efficacy and are still under study [10].

Thus, the authors agree with the conclusions of Fanaroff et al. and Don et al. that in cases with neonatal damages (e.g., encephalopathy) due to FIRS, there should be no attributable liability [50,61,62].

In cases of FIRS, the injuries of a newborn baby must be assessed to quantify a possible compensation [50,51]. FIRS can determine consequences to several systems (respiratory, cardiovascular, gastrointestinal and endocrinological), but the most relevant is brain damage with neurological impairments and a wide spectrum of possible disabilities [10,43,47,50,52,63]. Medical records of the child’s status at birth should be integrated with a detailed medicolegal examination.

The authors suggest that in the evaluation of permanent impairment, the medico-legal specialist must take into account the possible development of disabilities considering the patient’s age. Indeed, the compensation eligibility considers the long-term management of the patient’s disability, and the family’s psychological vulnerability in caring for severely sick children.

FIRS and placental infections are still relatively unknown conditions that are difficult to explain to families due to the complex etiopathogenesis. Numerous lawsuits result from a lack of appropriate communication between healthcare staff and families. In fact, families often seek legal advice to learn about what happened, rather than to obtain financial compensation. When adverse outcomes occur, physicians must discuss this with the family, addressing concerns and questions in a frank and open manner [50,60].

## 5. Conclusions

To conclude, the combination of current weaknesses in prenatal and postnatal diagnoses of FIRS together with the lack of proven methods to prevent or treat FIRS during pregnancy or postnatally, warrants more research and clinical attention.

All the medical specialists involved in FIRS management should be aware of their areas of vulnerability, which can inappropriately lead to malpractice litigation. They should always provide adequate clinical documentation and continuous interaction and communication with the families. The appropriate documentation of findings, a clear-cut explanation of medical decision-making, and periodic updating of standards of care are paramount in minimizing the liability risk.

## Data Availability

Not applicable.
